# Antibody Immunity to Zika Virus among Young Children in a Flavivirus-Endemic Area in Nicaragua

**DOI:** 10.3390/v15030796

**Published:** 2023-03-21

**Authors:** Omar Zepeda, Daniel O. Espinoza, Evelin Martinez, Kaitlyn A. Cross, Sylvia Becker-Dreps, Aravinda M. de Silva, Natalie M. Bowman, Lakshmanane Premkumar, Elizabeth M. Stringer, Filemón Bucardo, Matthew H. Collins

**Affiliations:** 1 Department of Microbiology, Faculty of Medical Science, National Autonomous University of Nicaragua, León 21000, Nicaragua; 2 Division of Infectious Diseases, Department of Medicine, Emory University School of Medicine, Atlanta, GA 30322, USA; 3 Department of Biostatistics, Gillings School of Public Health, University of North Carolina at Chapel Hill, Chapel Hill, NC 27599, USA; 4 Department of Family Medicine and Epidemiology, School of Medicine, University of North Carolina at Chapel Hill, Chapel Hill, NC 27599, USA; 5 Department of Microbiology and Immunology, School of Medicine, University of North Carolina at Chapel Hill, Chapel Hill, NC 27599, USA; 6 Division of Infectious Diseases, University of North Carolina at Chapel Hill, Chapel Hill, NC 27599, USA; 7 Department of Obstetrics and Gynecology, School of Medicine, University of North Carolina at Chapel Hill, Chapel Hill, NC 27599, USA

**Keywords:** Zika, antibodies, humoral immunity, neonatal immunity, flavivirus, antibody-dependent enhancement, passive immunity

## Abstract

**Objective:** To understand the dynamics of Zika virus (ZIKV)-specific antibody immunity in children born to mothers in a flavivirus-endemic region during and after the emergence of ZIKV in the Americas. **Methods:** We performed serologic testing for ZIKV cross-reactive and type-specific IgG in two longitudinal cohorts, which enrolled pregnant women and their children (PW1 and PW2) after the beginning of the ZIKV epidemic in Nicaragua. Quarterly samples from children over their first two years of life and maternal blood samples at birth and at the end of the two-year follow-up period were studied. **Results:** Most mothers in this dengue-endemic area were flavivirus-immune at enrollment. ZIKV-specific IgG (anti-ZIKV EDIII IgG) was detected in 82 of 102 (80.4%) mothers in cohort PW1 and 89 of 134 (66.4%) mothers in cohort PW2, consistent with extensive transmission observed in Nicaragua during 2016. ZIKV-reactive IgG decayed to undetectable levels by 6–9 months in infants, whereas these antibodies were maintained in mothers at the year two time point. Interestingly, a greater contribution to ZIKV immunity by IgG3 was observed in babies born soon after ZIKV transmission. Finally, 43 of 343 (13%) children exhibited persistent or increasing ZIKV-reactive IgG at ≥9 months, with 10 of 30 (33%) tested demonstrating serologic evidence of incident dengue infection. **Conclusions:** These data inform our understanding of protective and pathogenic immunity to potential flavivirus infections in early life in areas where multiple flaviviruses co-circulate, particularly considering the immune interactions between ZIKV and dengue and the future possibility of ZIKV vaccination in women of childbearing potential. This study also shows the benefits of cord blood sampling for serologic surveillance of infectious diseases in resource-limited settings.

## 1. Introduction

Zika virus (ZIKV) is a flavivirus primarily transmitted by *Aedes aegypti* mosquitoes, and it shares transmission ecology with the related flavivirus dengue (DENV) and the alphavirus chikungunya (CHIKV). ZIKV was first identified in Uganda in 1947, with very few cases reported in the 20th century [1]. Since 2007, outbreaks began to be recognized in Southeast Asia and Oceania [2], and retrospective studies have suggested an unappreciated endemicity in many Asian countries [3,4]. The 2007 outbreak on Yap Island in Micronesia demonstrated the ability of ZIKV to spread rapidly in a previously unexposed population. Serologic studies of this outbreak also revealed that (1) ZIKV can infect people with pre-existing immunity to DENV, and (2) ZIKV infection induces cross-reactivity to DENV and other flaviviruses. ZIKV caused a pandemic in 2015–2016, and autochthonous transmission has been reported in >80 countries, mostly in tropical regions [4]. More than one million people in the Americas have been infected by ZIKV, and more than 3700 confirmed cases of congenital Zika syndrome have been reported; ZIKV was declared a public health emergency of international concern in March 2016 [1]. Attack rates were high in certain regions, up to 73% in northeast Brazil [5], with seroprevalence now exceeding 50% in multiple areas, including our study site [6,7]. However, there are likely over one billion ZIKV-susceptible individuals living in areas where competent ZIKV vectors are endemic [8,9], making future ZIKV outbreaks highly likely in many regions. Recent outbreaks in India and ongoing, low-level transmission in several places, including the Americas, have been noted, which demonstrates the potential for additional epidemics and justifies the maintenance of robust surveillance systems and further development of countermeasures, such as vaccines [10].

While Zika is typically a self-limiting illness characterized by rash, fatigue, malaise, joint pain, headache, and conjunctivitis, it has been associated with severe complications such as Guillain–Barré syndrome [1]. The ZIKV pandemic also revealed that ZIKV can be sexually transmitted [11], a novel phenotype for a mosquito-borne virus that further complicates public health education and control efforts. ZIKV became notorious for its ability to cause severe congenital anomalies and a wide range of pathologies, collectively termed congenital Zika syndrome [12]. The affected babies of both symptomatic and asymptomatic mothers [13] suffered microcephaly, agenesis of the corpus callosum, anencephaly, lissencephaly, cerebellar hypoplasia, ventriculomegaly, cerebral calcifications, skull collapse, ocular disorders, and arthrogryposis [14,15]. It has also become clear that congenital ZIKV infection causes neurodevelopmental delays, affecting children with and without overt evidence of pathology at the time of birth [16,17]. Retrospective studies have identified the same neurologic sequelae and congenital defects in outbreaks prior to the emergence of ZIKV in the Americas [18,19]. However, the potential effect of ZIKV immunity passively acquired by babies from their mothers in utero on subsequent flavivirus exposure is not known. It has been described that a DENV infection occurring in the latter half of the first year of life can be exacerbated by low levels of residual DENV-reactive maternal antibodies (Ab) in young children that no longer confer protection [20,21].

In this study, we analyzed samples from an observational cohort that followed pregnant women and their children over two years, collecting serial serologic specimens for translational immunology and epidemiology studies in arbovirology. We specifically investigate the transfer efficiency and decay kinetics of ZIKV-specific Ab in two birth cohorts recruited during and soon after the ZIKV epidemic in León, Nicaragua. We hypothesized that the babies would acquire immunity to ZIKV according to the mother’s infection history but will become susceptible to ZIKV by the end of the first year of life. Addressing this hypothesis is important because it has been recently shown that ZIKV-induced Ab can exacerbate a subsequent DENV infection [22] and because a precise understanding of maternal-fetal humoral immune dynamics may inform the optimal implementation of ZIKV vaccines or other forms of immunoprophylaxis that are safe for pregnant women and children, which may become available in the near future [23,24].

## 2. Methods

### Study Design and Biospecimens

We enrolled pregnant women and their children in León, Nicaragua into two sequential cohorts during prenatal care at La Perla Maria Health Center or in Labor and Delivery at Hospital Escuela Oscar Danilo Rosales Argüello (HEODRA). The clinical and sociodemographic data at enrolment were obtained by abstraction from the medical record or participant-reported information via a standardized questionnaire. Further epidemiologic information was collected during follow-up visits, including questions about potential arbovirus exposure, symptoms, or confirmed diagnosis. These data were maintained in an electronic study database. Of 253 pregnant women (PW) enrolled in PW1 and 259 in PW2, 102 and 134, respectively, agreed to participate in longitudinal sampling (Table 1). The peak ZIKV transmission in Nicaragua occurred in July of 2016, and 60% of women in cohort PW1 were pregnant during the peak, with the remaining 40% becoming pregnant within the final 5 months of 2016 [25]. The earliest delivery in PW2 was November 15, 2017, which corresponds to conception in February 2017, a time when local ZIKV transmission had ceased. The inclusion criteria for the women included receiving prenatal care at La Perla Maria, delivering at HEODRA, and remaining in the area for the length of the study. At birth, maternal peripheral blood and cord blood samples were obtained. Blood samples were obtained from the children every three months from 3–24 months of age. A second maternal blood sample was obtained 24 months after delivery. The plasma was isolated by centrifugation and stored at −20 °C or −80 °C until tested. The plasma was heat-inactivated for 30 min at 56 °C prior to use in the serologic assays.

*Viruses and cells:* DENV WHO reference strains (DENV1 West Pac 74, DENV2 S-16803, DENV3 CH54389, and DENV4 TVP-360, initially obtained from Robert Putnak) and ZIKV H/PF/2013 were used [26]. Low-passage working stocks were prepared by infecting C6/36 *Aedes albopictus* mosquito cells (ATCC no. CRL-1660) at an MOI of 0.1 and harvesting the supernatant after 7 days. The amount of infectious virus in the supernatant was titrated on Vero *Cercopithecus aethiops* monkey cells (ATCC no. CCL-81) by quantifying focus-forming units (FFU) in a 96-well format [27,28]. The FFU per ml of supernatant was back-calculated from dilutions that resulted in ~50–75 foci (this range enables the most precise counting).

*ELISA:* ZIKV-binding IgG was measured by antigen-capture ELISA, as previously described [26]. The plates were coated with the flavivirus-reactive monoclonal antibody (4G2) in carbonate buffer (pH = 9.2) at a concentration of 100 ng per well. The plates were incubated at room temperature for 2 h or sealed overnight at 4 °C. The plates were blocked with 3% nonfat dry milk and incubated with ZIKV antigen derived from the supernatant of the C6/36 cell cultures for 1 h at 37 °C. The plasma was diluted to 1:100 and incubated at 37 °C for 1 h. The bound plasma IgG was detected by an alkaline phosphatase-conjugated goat anti-human IgG secondary Ab (Thermo Fisher, Waltham, MA, USA) and *p*-nitrophenyl phosphate substrate (Thermo Fisher, USA). Absorbance at 405 nm (optical density, OD) was measured by spectrophotometry. To detect ZIKV-specific IgG, the ZIKV EDIII ELISA was performed, as previously described [29].

*Neutralization Assays:* Neutralizing antibodies (NAb) were detected by a 96-well micro-focus reduction neutralization test (FRNT), as previously described [26]. The serial dilutions of plasma were mixed with approximately 75–100 focus-forming units of virus and incubated for 1 hr prior to infecting the Vero cell monolayer. After 40–72 h (incubation times are previously established based on the growth kinetics of each virus), the cells were fixed with MetOH/acetone, and the intracellular viral protein was detected by immunohistochemical staining with 4G2, which was detected by incubation with HRP-conjugated anti-mouse IgG (MilliporeSigma, St. Louis, MO, USA) and Trueblue substrate (VWR, Radnor, PA, USA). The foci were quantitated by semi-automated counting, and the NAb titers were calculated (GraphPad Prism 7). An abbreviated assay (eFRNT) was also used by running four four-fold dilutions of 1:20–1:1280 in singleton. The eFRNT value is a discrete number corresponding to the dilution factor at which 50% maximum FFU is observed or the average of the two dilution factors between which the 50% FFU threshold is crossed [25].

*Data management and statistical analysis:* The cutoff in ELISA was calculated for each plate as the average OD of the negative control plasma + 3 *standard deviation + 0.1 (correction factor for a more conservative cutoff). The FRNT50 values were determined by interpolation from a fit curve (four-parameter, sigmoidal dose-response, variable slope, Prism 7). The FRNT50 values were required to have an R^2^ > 0.75, a Hill slope > 0.5, and an FRNT50 falling within the range of the dilution series. Where Pearson correlation analyses were performed, the R^2^ and *p* values are reported. The longitudinal ELISA data were graphed by spaghetti plots to show the individual trajectories over time and plotted as a ratio of the OD to the lower limit of detection. Although the study design stipulated precise sampling times at three-month intervals through 24 months of age, we were able to use the exact dates of birth and sample acquisition to support a more robust and time-resolved analysis. Thus, time is designated as “weeks from birth” for these analyses. A composite decay curve was generated by fitting longitudinal mixed-effects models to the data with random effects for each individual subject. Two models were fit to each set of antibody data from each cohort, one assuming the antibody titer decayed exponentially, and the other modeling the titer over time as a quadratic polynomial.

## 3. Results

### 3.1. Seroprevalence of Maternal Flavivirus Cross-Reactive and ZIKV-Specific IgG in Cord Blood

**Figure 1:**
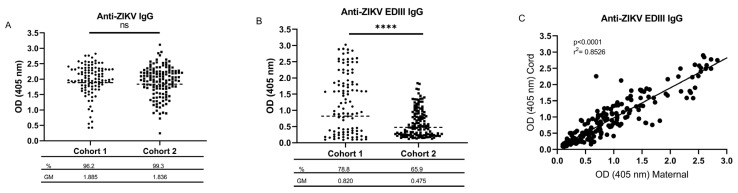
Seroprevalence of ZIKV-reactive IgG in cord blood. ZIKV IgG (**A**) and ZIKV EDIII (**B**) ELISA were performed on cord blood plasma diluted 1:200 from Cohort 1 (*n* = 104) and Cohort 2 (*n* = 135). The cutoff for positivity was determined on each plate by running negative control plasma. The percent seropositivity for each assay is shown beneath the cohort names, and the geometric mean (GM) of OD for positive samples is shown beneath that. (**C**) The OD value from the ZIKV EDIII ELISA for available paired samples (*n* = 102) from mother peripheral blood and cord blood are graphed in a scatter plot to assess the efficiency of ZIKV-reactive IgG transfer across the placenta. Pearson correlation analysis was performed, and the R^2^ and p values are included in the upper left corner of each graph. ns, not significant; ****, *p* < 0.0001 according to an unpaired Student’s t-test comparing differences between GM of Cohort 1 vs. Cohort 2.

We first asked whether ZIKV-specific IgG was detectable in the cord blood samples obtained at birth in both cohorts, expecting a high rate of positivity reflecting the intense transmission of ZIKV in the study site just prior to enrollment beginning for the first cohort. The positivity rate among the cord blood samples tested by ZIKV IgG ELISA was 96.2% and 99.3% for Cohort 1 and Cohort 2 (Figure 1A), consistent with the high DENV endemicity in this region and the detection of cross-reactive flavivirus antibodies (Abs) by this assay [25]. The testing by the more specific ZIKV EDIII ELISA [22,29,30] indicated 82 of 104 (78.8%) cord blood samples in Cohort 1 and 89 of 135 (65.9%) samples in Cohort 2 were positive (Figure 1B). This result corresponds to 82 of 102 (80.4%) mothers in Cohort 1 (two sets of twins) and 89 of 134 (66.4%) mothers in Cohort 2 (one set of twins). A higher geometric mean (GM) OD was noted for the samples testing positive in the ZIKV EDIII assay for Cohort 1 compared to the Cohort 2 samples (1.281 vs. 0.735, *p* < 0.0001, Student’s t-test). No significant difference was observed for the mean OD in the ZIKV ELISA between the two cohorts. As an additional validation that the ZIKV EDIII ELISA was detecting ZIKV-specific Ab, we checked for ZIKV-NAb in six samples with a high OD in the ZIKV EDIII assay. As expected, the samples consistently exhibited elevated levels of ZIKV-NAb (Supplementary Figure S1).

### 3.2. Kinetics and Composition of Maternal-Derived ZIKV-Reactive Antibodies

**Figure 2:**
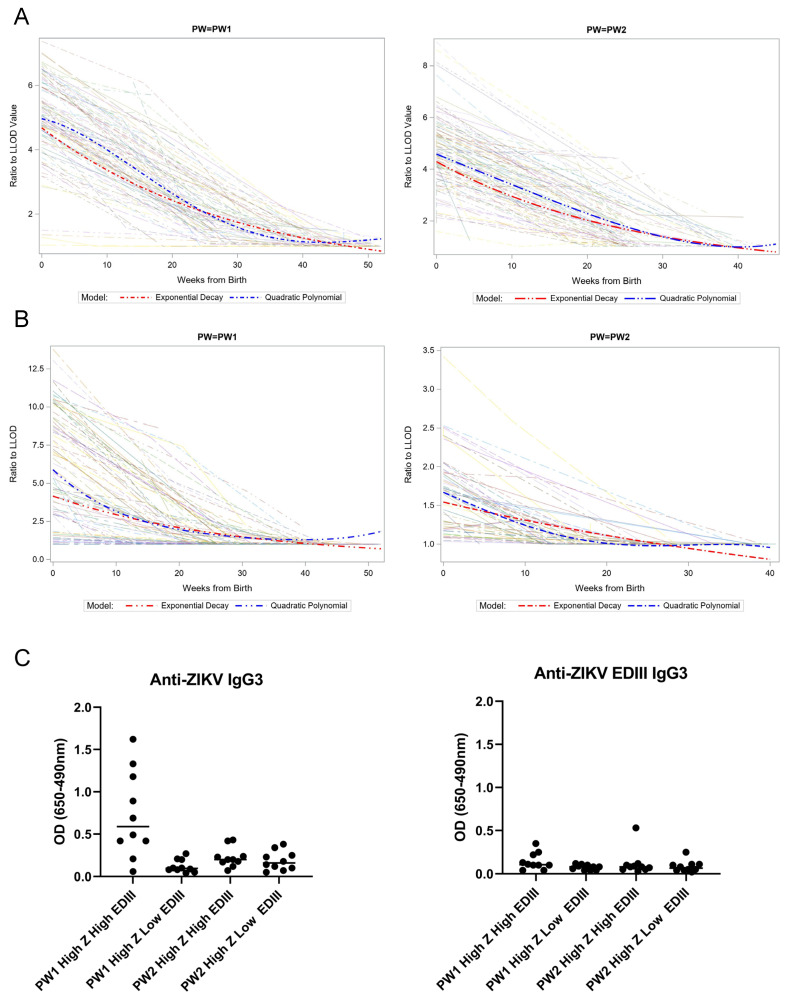
ZIKV-reactive antibody kinetics and IgG subtype. Decay of IgG reactive to ZIKV (**A**) and ZIKV EDIII (**B**) is shown for infants in both PW1 and PW2 cohorts. Spaghetti plots of trajectories were generated from the OD from each ELISA as a ratio to the LLOD, using a continuous time scale of weeks of life for each infant. Data from individual infants are shown as semi-transparent lines. Fit lines summarizing all data were calculated using an exponential decay (red dashed line) and a quadratic polynomial (blue dashed line) model. (**C**) IgG3 reactivity to ZIKV (left panel) and ZIKV EDIII (right panel) is shown in a subset of cord blood samples (*n* = 10 per subgroup) from subgroups indicated on the *x*-axis. OD, optical density; LLOD, lower limit of detection. X-axis legend of left graph of panel C: needs space between LOW and EDIII in the 2 group from left.

A primary objective of this study was to assess the kinetics of ZIKV-specific immunity in babies born to mothers with a prior ZIKV infection. This analysis was designed to be conducted relative to the starting levels of anti-ZIKV EDIII, so we first approximated the efficiency of IgG transfer across the placenta by performing ELISA on the matched samples from the maternal peripheral blood and the cord blood. We previously found a strong correlation between the OD values from the maternal peripheral blood at birth compared to the cord blood for the ZIKV IgG ELISA [25]. We confirmed a similarly strong correlation for the ZIKV EDIII ELISA (R^2^ = 0.8526, *p* < 0.0001, Pearson correlation; Figure 1C). We then measured the ZIKV-reactive Ab levels in the children over the first 12 months of life at three-month intervals. In both cohorts, the Ab levels detected by both assays became undetectable between 6 and 9 months after birth (Figure 2A,B). The rate of decay for the exponential model was estimated to be 3.2% and 3.9% decay per week in the first year of life for the ZIKV antibody in PW1 and PW2, respectively, and 3.4% and 1.6% decay per week for the EDIII antibody in PW1 and PW2, respectively. A similar trend was shown by the quadratic polynomial model. Prior work found that anti-ZIKV Ab of the IgG3 subtype were significantly elevated early after ZIKV infection and may represent a viable target for specific serologic diagnosis of a recent ZIKV infection [5]. ZIKV-reactive IgG3 subtype antibodies were selectively detected in the ZIKV IgG ELISA among infants who were ZIKV EDIII+ and born soon after the peak local ZIKV transmission (PW1), suggesting that the composition of IgG subtypes transferred to babies is dynamic, depending on the recent infection history (Figure 2C). IgG3 binding to ZIKV EDIII was not detected in any of the subgroups tested.

### 3.3. Incident Dengue Infection after Maternal-Derived Antibody Decay

**Figure 3:**
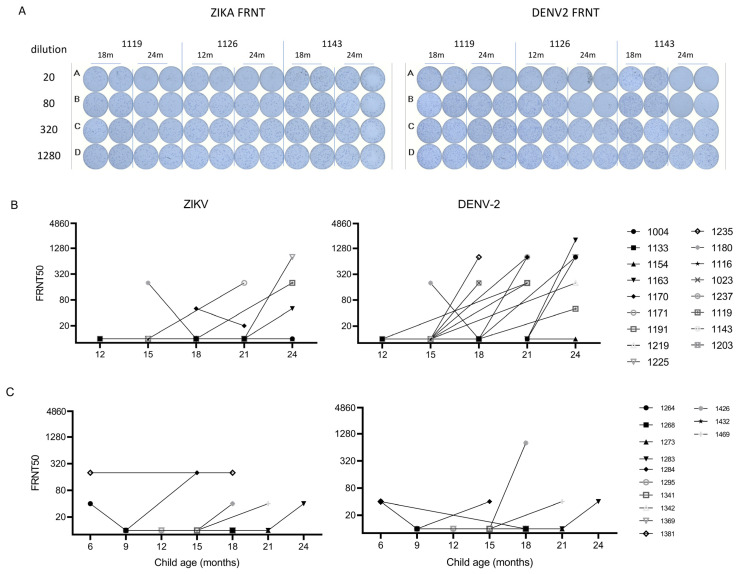
Incident flavivirus infection detected after initial decay of maternal-derived anti-ZIKV IgG in early life. Paired samples available from time points before and after the observed increase in anti-ZIKV IgG were selected for NAb testing. (**A**) Representative raw FRNT data measuring NAb to ZIKV (left) and DENV2 (right) are shown for three subjects. (**B**,**C**) eFRNT50 values for ZIKV (left) and DENV2 (right) are shown for suspected cases of incident flavivirus infection in Cohort 1 (**B**) and Cohort 2 (**C**). For nine samples with suspected incident flavivirus infection., and eFRNT50 values for paired samples are linked by a solid line.

Interestingly, after testing all the longitudinal samples available for children through two years of age, we observed that ZIKV IgG levels in 43 of 343 (13%) children persisted >9 months or increased (by ≥0.3 OD) after the initial decay (Supplementary Figure S2), suggestive of a congenital or incident flavivirus infection. We hypothesized this finding was indicative of an interval flavivirus infection. To distinguish between ZIKV- and DENV-induced Ab production, we selected two sequential samples flanking, or after the time of, the suspected flavivirus infection and tested for NAb against ZIKV and DENV2 (because a large DENV2 outbreak occurred in 2018–2020 in Nicaragua [22]). We tested samples from 17 participants in PW1: 16 exhibited a ≥0.5 OD increase in anti-ZIKV IgG, and one had persistent anti-ZIKV IgG (1203). There were 13 participants in PW1 with an increase in OD between 0.3 and 0.5, who were not tested for NAb due to limited resources. Of the 17 participants tested in PW1, 4 had ZIKV-neutralizing Abs that increased at later time points, and 11 exhibited an increase in DENV2 NAb. Of the ZIKV NAb+, two had DENV2 NAb ≥ four-fold higher, consistent with incident DENV2 infection with lower levels of cross-reactive Ab to ZIKV, and two had ZIKV and DENV2 NAb in the same range, consistent with a flavivirus infection that could not be further distinguished with the existing data. Among the 13 participants from PW2, all exhibited a ≥ 0.3 OD increase. One (1426) of the 13 participants tested in PW2 also exhibited clear DENV2 NAb seroconversion (Figure 3, Supplementary Figure S3). For the participants in PW1, we have additional information on the ZIKV infection status of mother–baby dyads, including a single confirmed case of congenital ZIKV infection (1203, summarized in Supplementary Table S1) [25].

## 4. Discussion

In this study, we found that ZIKV-specific Ab responses were detectable over the first several months of life of children born to mothers in a flavivirus-endemic region of Nicaragua following the emergence of ZIKV. As expected, most of the women tested positive with the ZIKV ELISA because of the nearly 100% DENV seroprevalence [25,31] and cross-reactivity to ZIKV in DENV-immune subjects [28,32]. However, using an ELISA more specific for ZIKV [29] yielded a seroprevalence of ~65–75%, slightly higher than prior estimates in this region [7,25] and consistent with the seroprevalence following intense ZIKV outbreaks in other places [2,5]. These data demonstrate that the ZIKV EDIII ELISA is a reliable measure of ZIKV-specific immunity into late convalescence. The value of specific assays capable of distinguishing immunity induced by a prior infection with one or more related flaviviruses is difficult to overstate. Billions of people live in regions endemic for two or more flaviviruses. The antigenic relatedness within this genus is problematic in that it may compromise the specificity of serologic assays [32,33,34,35], which constitute common tools employed for epidemiologic surveillance, diagnosis, and outcome measures in clinical research [36]. Since the emergence of ZIKV in the Western Hemisphere in 2015, several interesting and innovative methods have been reported with the ability to distinguish DENV- vs. ZIKV-specific Ab indicative of recent or remote infections by that virus [37,38,39,40,41,42,43,44]. A thorough discussion of the advantages and limitations of each assay is beyond the scope of this article. Suffice it to say that the ZIKV EDIII assay used in this study proved useful in identifying ZKV-specific immunity in a background of predominantly multitypic DENV immunity. This same assay recently demonstrated utility in a large epidemiologic study in the Philippines [29], and in identifying the risk of severe outcomes in DENV infection among the study participants sampled in the recent DENV2 outbreak in Nicaragua [22]. Finally, this assay has advantages, including the use of a recombinant protein subunit antigen that can be produced at a large scale, a simple detection protocol amenable to assay deployment in resource-limited settings, and the potential to be integrated into multiplex assays.

We also demonstrated a strong correlation between ZIKV EDIII IgG in maternal and cord blood, as observed previously for ZIKV IgG [25]. Our data indicated a ZIKV prevalence in our study population on par with that in other areas determined with traditional sampling approaches obtaining peripheral blood [2,5,7]. Cord blood testing is attractive as a robust and cost-efficient approach for accomplishing epidemiologic surveillance at scale. Cord blood is typically a discarded specimen following delivery, and obtaining cord blood confers no risk to the mother or newborn after the cord clamping. Additionally, pregnant women from all socioeconomic strata and locations of residency consistently access the healthcare system, making this group a good surrogate sample for the general population (with imbalances in age, sex, occupational exposure, etc., noted). Thus, this study, along with our prior work [16,25], demonstrates that serologic detection of maternal Abs in cord blood can provide reliable information about the flavivirus infection history in an individual and population.

Analyzing the kinetics of passively-acquired flavivirus immunity in young children, we found that ZIKV-reactive IgG decays at an estimated rate of 3.23% and 3.85% decay per week to low or undetectable levels by 6 to 9 months of age, confirming recent findings of Coutinho, et al. that IgG declined independently of anti-ZIKV IgM detection, and similarly in babies with and without known congenital ZIKV infections in Brazilian and Nicaraguan infants [45]. The Coutinho study had robust criteria for constructing its two cohorts, with Cohort 1 being infants born to mothers with RT-PCR-confirmed ZIKV infections, but it did not further discern ZIKV-specific Ab responses from cross-reactive Ab elicited by prior DENV. Our paper provides the added value of detecting ZIKV-specific Ab (via NAb testing and the ZIKV EDIII ELISA) with similar kinetics as the non-specific anti-flavivirus Ab detected by the ZIKV IgG ELISA [46]. Interestingly, there were unexpected increases in ZIKV-reactive Ab in a few children after the passively-acquired maternal IgG had decayed to undetectable levels. Informed by the public knowledge of a DENV-2 outbreak in 2019 [22], we confirmed by NAb testing that this ZIKV-cross-reactive Ab was elicited by DENV-2 infections in several children. Another crucial finding was that IgG3 was only detected among the Ab measured by the ZIKV ELISA in Cohort 1 in ZIKV EDIII+ participants. IgG3 is an understudied IgG isotype that accounts for <10% of total IgG and has distinct structural features and glycosylation states compared to other isotypes, potentially conferring greater capacity to drive Fc-mediated effector functions or neutralization [47]. Additional study of the distribution and specificity of Ab subtypes present in mothers and newborns is warranted. The factors that determine the complement of Ab immunity inherited by the fetus in utero due to the trans-placental movement of immunoglobulins remain a controversial active area of study [48,49,50]. IgG3 has been described as an important mediator of immunity and viral clearance of chikungunya [51], another *Aedes*-borne virus. These results are a reminder that the maternal immune response evolves over time, and Abs with different specificities and effector functions may be differentially present at various time points following infection.

The strengths of this study include its prospective design and longitudinal sampling, achieving a large sample size with excellent retention. Having corroborating data from multiple robust serologic assays to bolster our conclusions was another asset. It was also reassuring that the prevalence of ZIKV infection was similar across two independent samples obtained from the same population at two different times. Moreover, the attack rate of ZIKV, as determined by ZIKV EDIII positivity, was in the same range as the measurements made by different serologic methods, performed at our study site and other places that experienced intense ZIKV transmission. There were also important limitations. The low volume of samples from infants precluded more extensive testing, such as determining the NAb titers in multiple flaviviruses. It would have been interesting to assess the relationships of relative NAb titers among ZIKV and the four DENV serotypes, as we have done previously [52,53], or see whether the seroprevalence of CHIKV infections was concentrated within the ZIKV EDIII positive participants. Since CHIKV is an alphavirus that shares the *Aedes aegypti* mosquito vector but does not induce cross-reactive Ab to ZIKV, a significantly increased likelihood of being co-positive for these two viruses would identify those likely to experience the most extensive mosquito exposure. We were unable to fully assess the relative representation of the IgG subclasses, Fc-mediated effector function, or IgG glycosylation states within ZIKV-specific Abs, which are implicated in placental transfer and anti-viral immunity [49,54,55]. While our study captured multiple incident DENV2 infections, a larger prospective cohort would be required to determine the relative risk of DENV infection based on the infant serostatus. That said, we do demonstrate plausibility for severe dengue in young children of ZIKV-immune mothers, given the observations of severe dengue in this age group as maternal-derived DENV NAb decay [56] and a recent study showing that ZIKV infection can exacerbate subsequent DENV infection [22].

Further work is needed. It will be important to understand when babies born to ZIKV-immune mothers become susceptible to ZIKV infection, which may inform the optimal scheduling of ZIKV vaccines. Ideally, a simple assay such as the ZIKV EDIII ELISA would provide a quantitative cut-off for protective immunity. While NAb are widely thought to be critical components of protective immunity, and recent studies support the potential to define a threshold of NAb that indicates protection, there is no correlate of protection for ZIKV that has been defined in humans [57,58,59]. It remains unclear whether the levels of ZIKV EDIII-binding Ab could define protection. Recently, it was found that NAb activity in polyclonal serum did not comprise a major contribution by ZIKV EDIII-binding Ab [60], similar to previous observations with DENV [61]. However, the EDIII-binding Ab may identify a state of protective immunity to ZIKV without direct correlation to the NAb titers.

Given that this study focuses on young children, it is important to consider that the endogenous serologic response arising from fetal or neonatal ZIKV infection may be shorter-lived or qualitatively different when compared to adults. Our understanding of adaptive immune responses in the fetal and neonatal period remains in a fledgling state. Just as the mother must tolerate the allogeneic fetus for a successful pregnancy, the fetus must avoid excessive immune activation to alloantigens from the mother. Thus, a tolerogenic environment, largely mediated by the regulatory T cells, predominates at the earliest stages of life as the constituent cells of the adaptive immune system are beginning to appear and mature. An epigenetic restriction of effector molecule expression is also heightened in newborns compared to adults. Several factors may limit the induction of a durable Ab response to infection or vaccination in early life. IgM is the Ab isotype produced constitutively and in higher abundance in early life, while class-switching to isotypes, such as IgG, is typically associated with adaptive immune memory. The infrastructure for secondary lymphoid structures, where high-affinity B cell clones can be iteratively selected, is not fully developed. There is an impaired T follicular helper cell function to support B cell activation and inadequate niches in the bone marrow to sustain long-lived plasma cells. Thus, there are myriad features of the nascent immune system that may alter the quality and durability of humoral immunity in early life [62]. We know that up to 40 babies in Cohort 1 had in utero ZIKV exposure [25], but only two babies (1119 and 1126) had detectable NAb to ZIKV after 9–12 months. The lack of durable ZIKV NAb in an infant with a confirmed congenital infection (1203) indicates that long-term humoral immunity may not be generated by a virus infection of the fetus. For 1126, the estimated level of NAb to ZIKV was ≥ four-fold lower than that of DENV2, suggesting a recent DENV2 infection and lower-level cross-neutralization of the ZIKV. For 1119, there is clearly a seroconversion between 18 and 24 months, but the relative levels of NAb to ZIKV compared to DENV2 were not sufficiently different to deduce the infecting virus. Thus, more work should be done to fully characterize multiple immune parameters in natural history studies.

## 5. Conclusions

In conclusion, this study provides critical information and a framework for evaluating flavivirus epidemiology and immunologic risk for dengue in children living in flavivirus-endemic areas. Furthermore, systematic testing of cord blood represents a promising but underutilized tool for emerging infection surveillance [46]. Finally, the array of assays employed here can support future work to track virus-specific immunity and monitor for potential pathogenic Ab signatures in newborns born to mothers infected by or vaccinated against ZIKV. Given the ongoing efforts to develop ZIKV vaccines and monoclonal antibodies with therapeutic and prophylactic indications, and the potential immune interaction between DENV and ZIKV, expanding research efforts such as this study is crucial to inform clinical trial design in flavivirus-endemic areas and the eventual implementation of interventions such as vaccines—particularly among pregnant women [63].

## Figures and Tables

**Table 1:** Clinical and sociodemographic data of cohorts studied.

Mother’s Characteristics	No. of Subjects (%) ^a^	
	Cohort 1 *n* = 102	Cohort 2 *n* = 134
**Delivery dates (range)**	5 February 2017 to 9 October 2017	15 November 2017 to 4 July 2018
**Mean mother’s age at delivery (year) (±SD)**	23.8 (±7.1)	24.1 (±5.5)
** Mean gestational week at birth (week) (±SD) **	38.4 (±1.9)	38.6 (±1.4)
** <37 weeks **	10 (10%)	12 (9%)
**Cesarean delivery**	47 (46%)	56 (42%)
**Previous diagnosis of chikungunya**	39 (38%)	54 (40%)
**Previous diagnosis of dengue**	7 (7%)	12 (9%)
**Previous diagnosis of Zika**	3 (3%)	1 (1%)
**Children’s characteristics at birth**		
**Gender ^b^**		
**Female**	49 (47%)	78 (58%)
**Male**	55 (53%)	57 (42%)
**Mean head circumference (cm) ±SD**	33.9 ± 1.5	33.9 ± 1.6
**Mean child length (cm) ±SD**	49.8 ± 3.2	50.1 ± 2.6
**Mean birth weight (g) ±SD**	3087 ± 464.8	3102 ± 488.8
**Birth defects ^c^**	2 (2%)	2 (1%)
**Microcephaly**	1 (1%)	---
** Household characteristics **		
** Brick wall **	79 (78%)	114 (85%)
** Municipal piped water supply **	76 (75%)	114 (85%)
** Indoor toilet **	62 (61%)	88 (66%)
** Cement or ceramic floor **	62 (61%)	82 (61%)
** Electricity **	97 (95%)	133 (99%)

^a^ Data represent no. (%) of study subjects, unless otherwise specified.

^b^ Includes 2 sets of twins in Cohort 1 and 1 set of twins in Cohort 2; then total children in Cohort 1 = 104, and Cohort 2 = 135.

^c^ Includes microcephaly, agenesis of corpus callosum, spina bifida, and anencephaly.

## Data Availability

All the relevant data supporting the conclusions reached in this study are published in this article, including the raw data for the ELISA and neutralization assays on a subset of samples selected for in-depth analysis of incident flavivirus infection. The complete raw data set including the OD values for all the ELISA testing can be shared upon request.

## Supplementary Materials

The following supporting information can be downloaded at: https://www.mdpi.com/article/10.3390/v15030796/s1, Figure S1: Longitudinal ZIKV ELISA data for children with persistent or resurgent ZIKV IgG; Figure S2: Determination of infecting flavivirus by neutralizing antibody testing; Figure S3: Determination of infecting flavivirus by neutralizing antibody testing; Table S1: Information on suspected incident flavivirus cases.
