# Supplementary material for: Antibody Immunity to Zika Virus among Young Children in a Flavivirus-Endemic Area in Nicaragua
Source: Viruses. 2023 Mar 21;15(3):796. doi: 10.3390/v15030796 (PMC10052059; doi:10.3390/v15030796)
Supplement: Supplementary file 1 [file viruses-15-00796-s001.zip › viruses-2256452-supplementary.pdf]

Supplementary Material

Antibody immunity to Zika virus among young children in a flavivirus-endemic area in Nicaragua

Supplementary Table S1. Information on suspected incident flavivirus cases

Study Code	ZIKV Infection Status	Comment
1004	No ZIKV infection	
1023	ZIKV immune	
1027	ZIKV immune	
1116	ZIKV infection during pregnancy	Mom ZIKV FRNT50 ↑ by >3-fold during pregnancy
1119	ZIKV immune	
1133	-	Not included in study
1143	No ZIKV infection	
1154	No ZIKV infection	
1163	-	Not included in study
1170	ZIKV immune	
1171	-	Not included in study
1180	ZIKV immune	
1191	-	Not included in study
1203	ZIKV infection during pregnancy	Congenital infection confirmed by cord ZIKV IgM+
1219	ZIKV immune	
1225	ZIKV immune	
1235	No ZIKV infection	
1237	ZIKV immune	

Figure S1. ZIKV-neutralizing antibodies are detected in samples positive by ZIKV EDIII ELISA. A-B) Kinetics of ZIKV-binding (A) and ZIKV-neutralizing (B) antibodies are shown over the first year of life for select ZIKV EDIII+ samples. Linked ELISA and eFRNT result are shown for each sample at birth (cord blood; C) and 3 months (D). ZIKV-neutralizing antibodies are consistently detected the same samples reactive by ZIKV EDIII ELISA, corroborating the interpretation of prior ZIKV infection in the mothers of these babies.

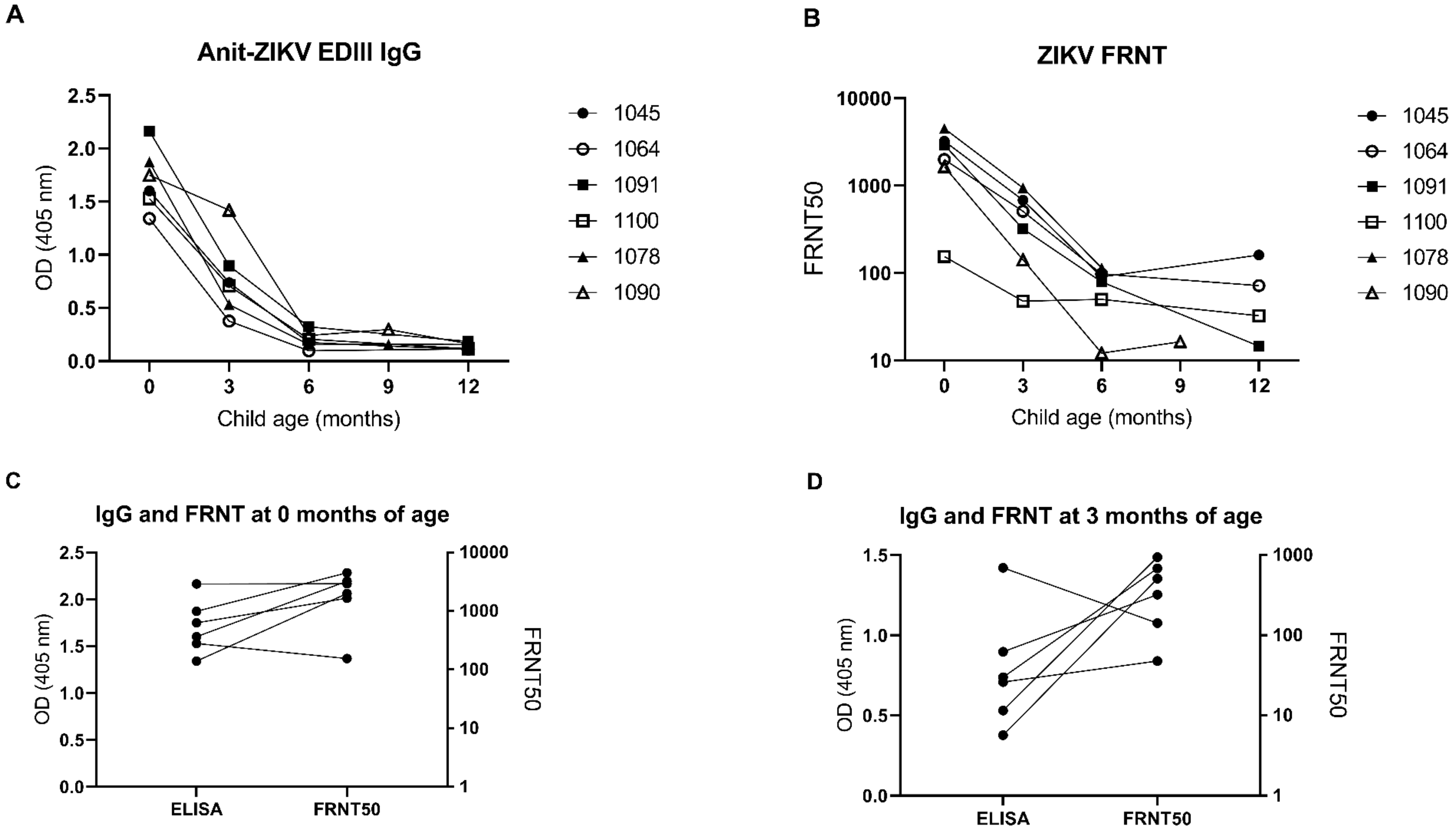

Figure S2. Longitudinal ZIKV ELISA data for children with persistent or resurgent ZIKV IgG. Raw optical density (OD) values are shown for longitudinal samples, grouped by participant code on x-axis with time points indicated by numerals (months of age, 0=cord blood), from children in PW1 cohort (A) and PW2 cohort (B). Samples with an increase of 0.5 OD are shown for PW1 and an increase of 0.3 OD for PW2. The lower threshold was used to select samples for further testing in PW2 because there were many fewer examples of suspected incident flavivirus infection. PW1 samples with an increase of 0.3 were noted but were not tested for neutralizing antibodies due to limited resources.

A

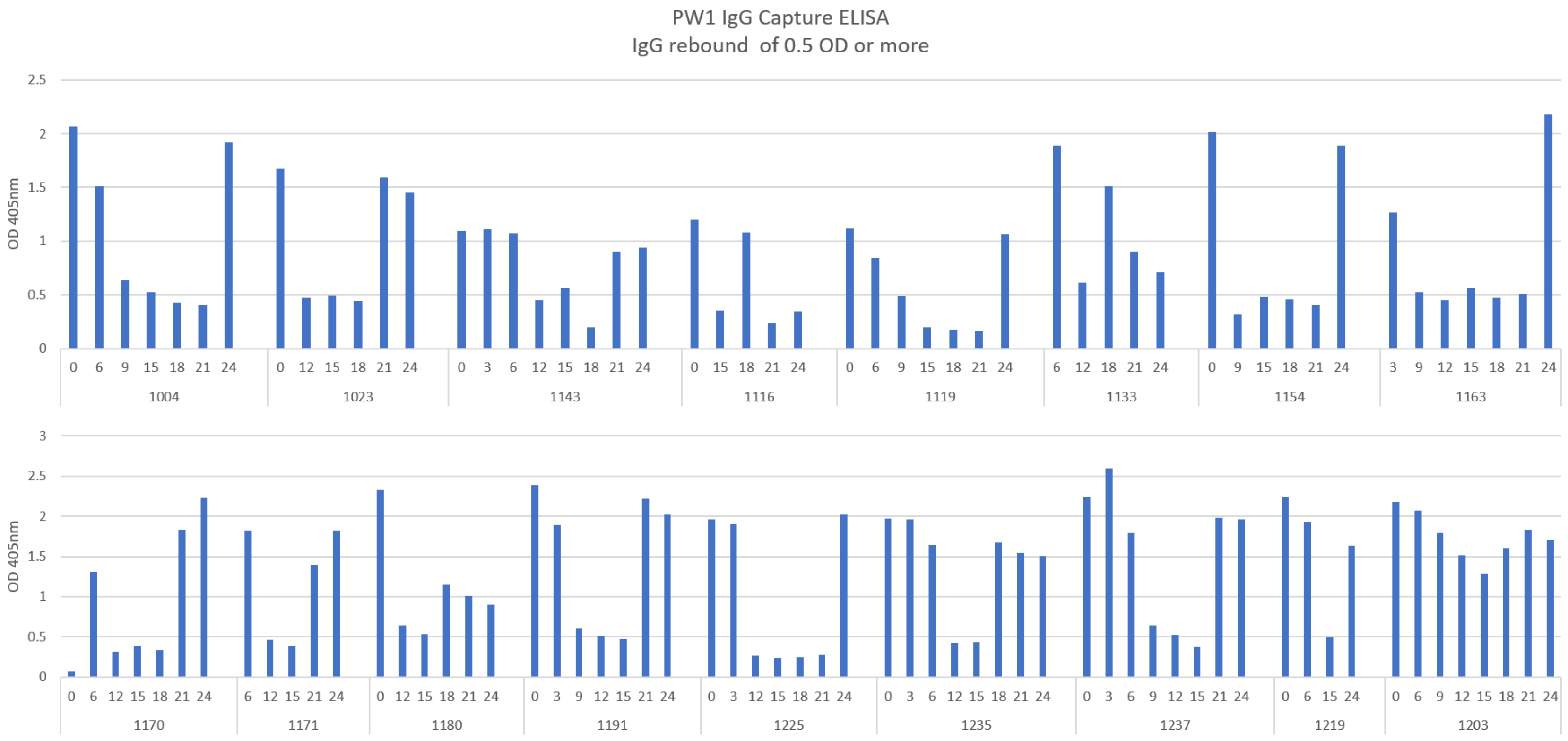

B

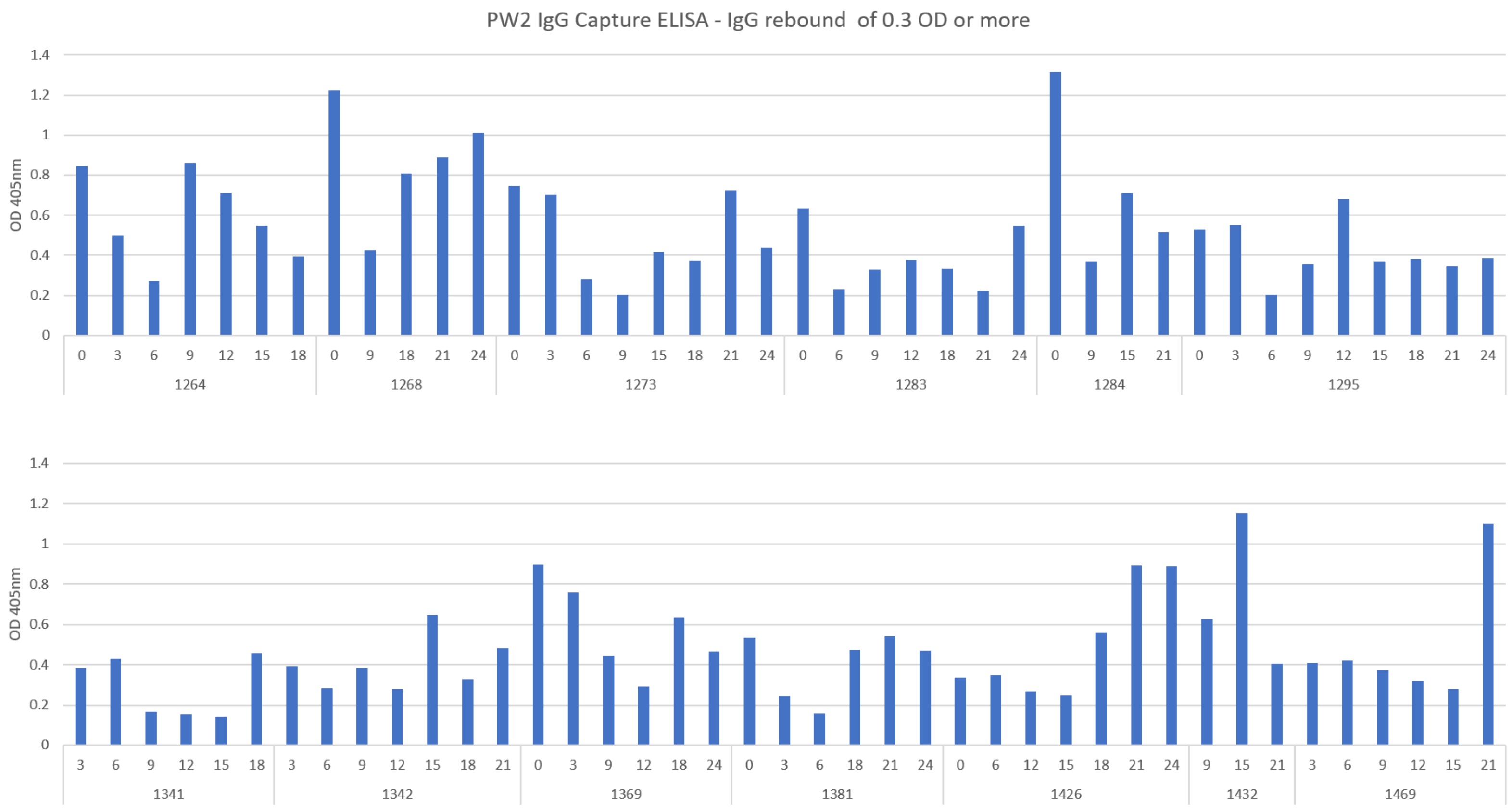
PW2 IgG Capture ELISA - IgG rebound of 0.3 OD or more
OD 405nm
1264
1268
1273
1283
1284
1295
1341
1342
1369
1381
1426
1432
1469

Figure S3. Determination of infecting flavivirus by neutralizing antibody testing. For the subset of samples identified by IgG ELISA, neutralizing antibody (NAb) titers were estimated by eFRNT at time points before and after the observed increase in OD (Figure S1). Raw data are shown consisting of focus staining for the indicated virus (ZIKV or DENV2) at the indicated time point for each sample. The dilution factor is shown at the left of each image (four 4-fold dilutions ranging 1:20 to 1:1280). Technical replicates were performed for all samples except for 1023, 12-3, 1119, and 1143, which were run in singleton.

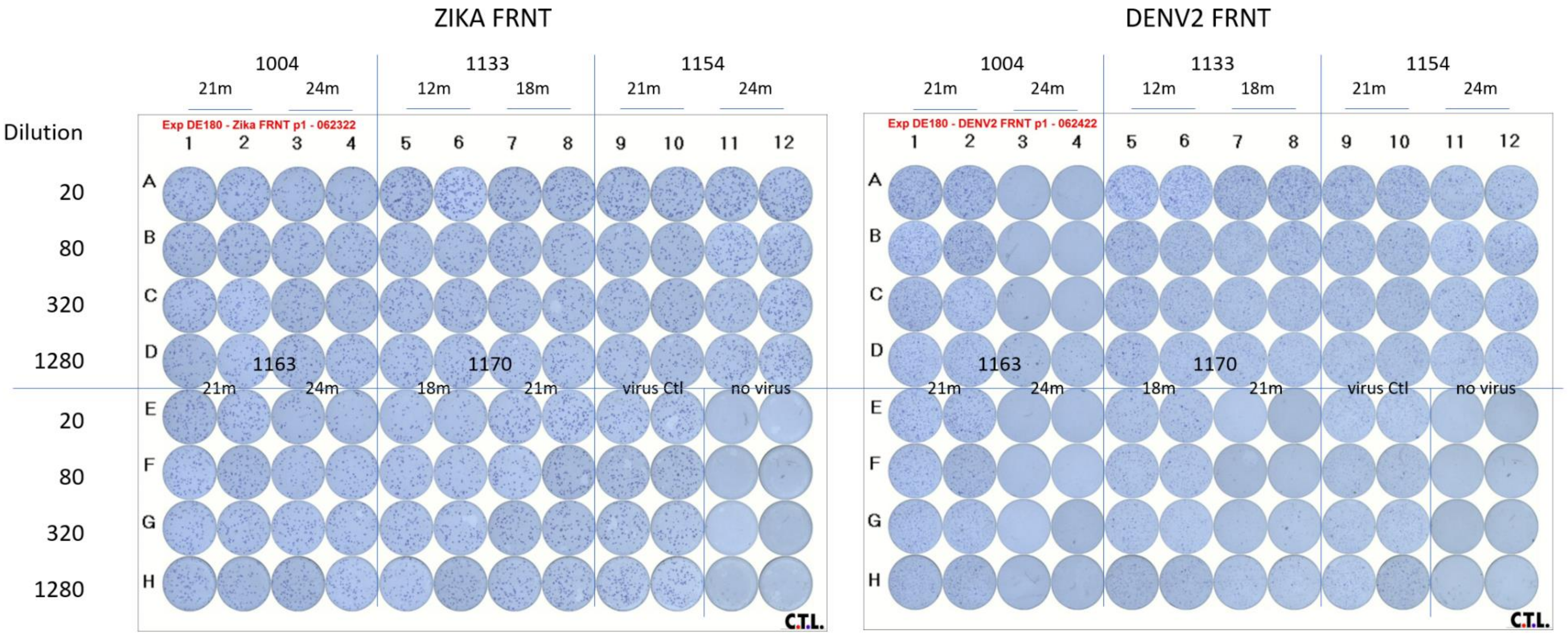

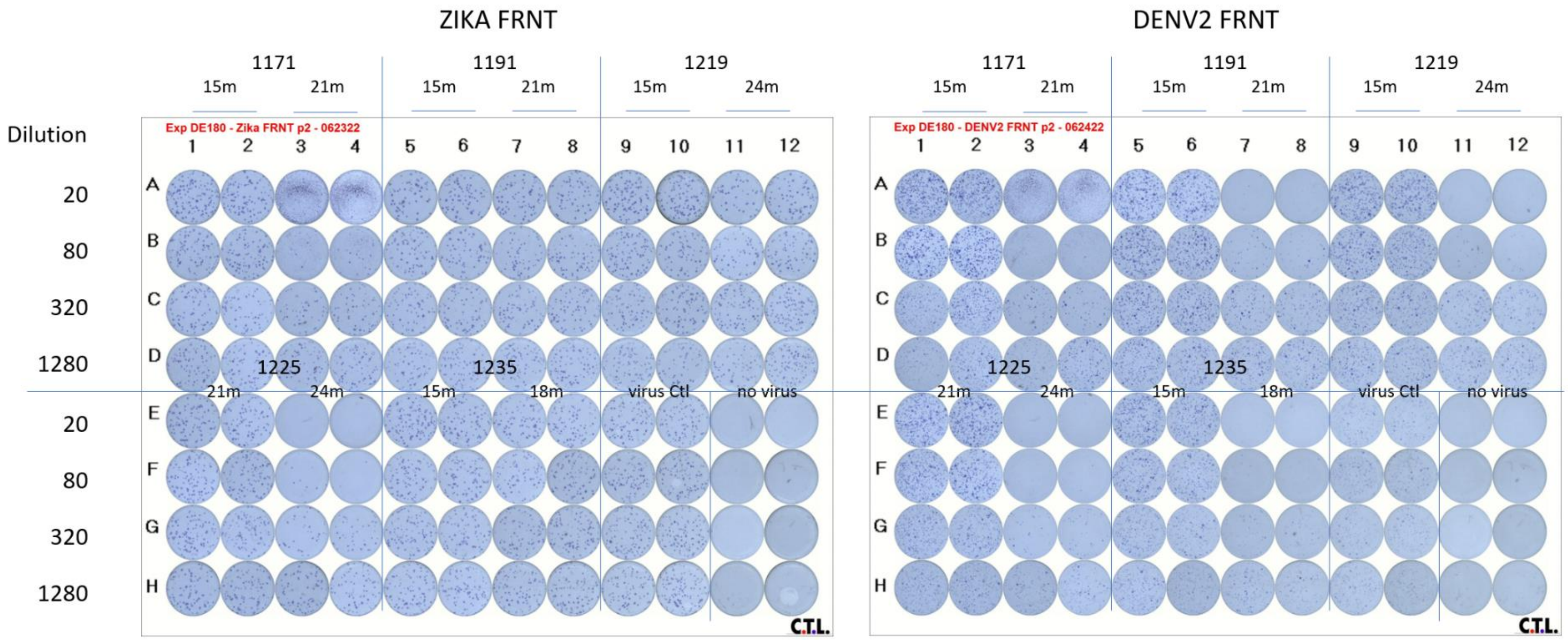
ZIKA FRNT
DENV2 FRNT
1171
15m
21m
1191
15m
21m
1219
15m
24m
Dilution
20
80
320
1280
20
80
320
1280
Exp DE180 - Zika FRNT p2 - 062322
Exp DE180 - DENV2 FRNT p2 - 062422
1225
21m
24m
1235
15m
18m
virus Ctl
no virus
C.T.L.

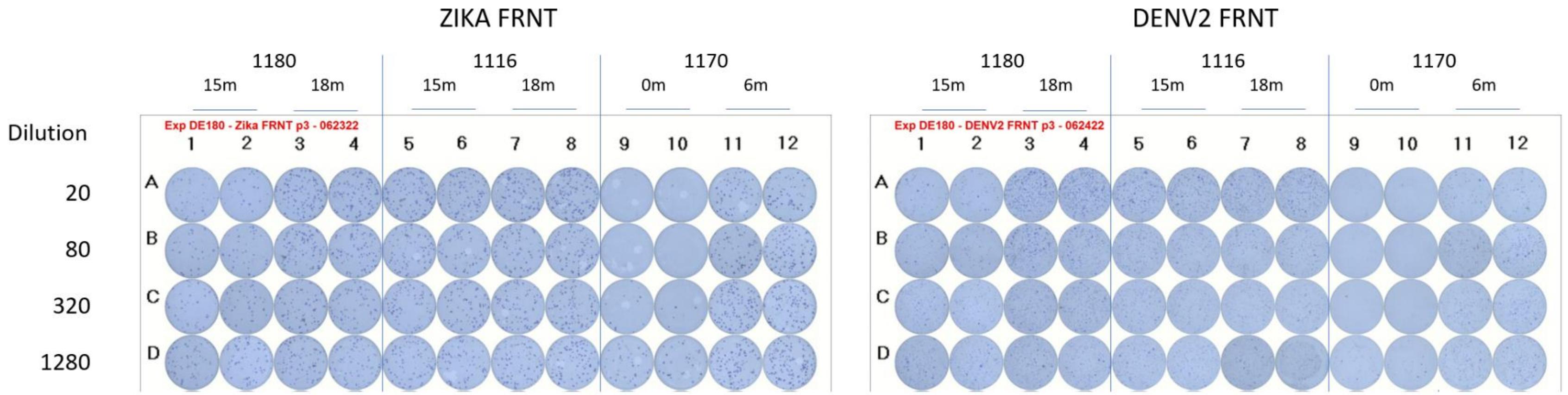

ZIKA FRNT
DENV2 FRNT
1180
1116
1170
15m
18m
0m
6m
Dilution
20
80
320
1280
Exp DE180 - Zika FRNT p3 - 062322
Exp DE180 - DENV2 FRNT p3 - 062422

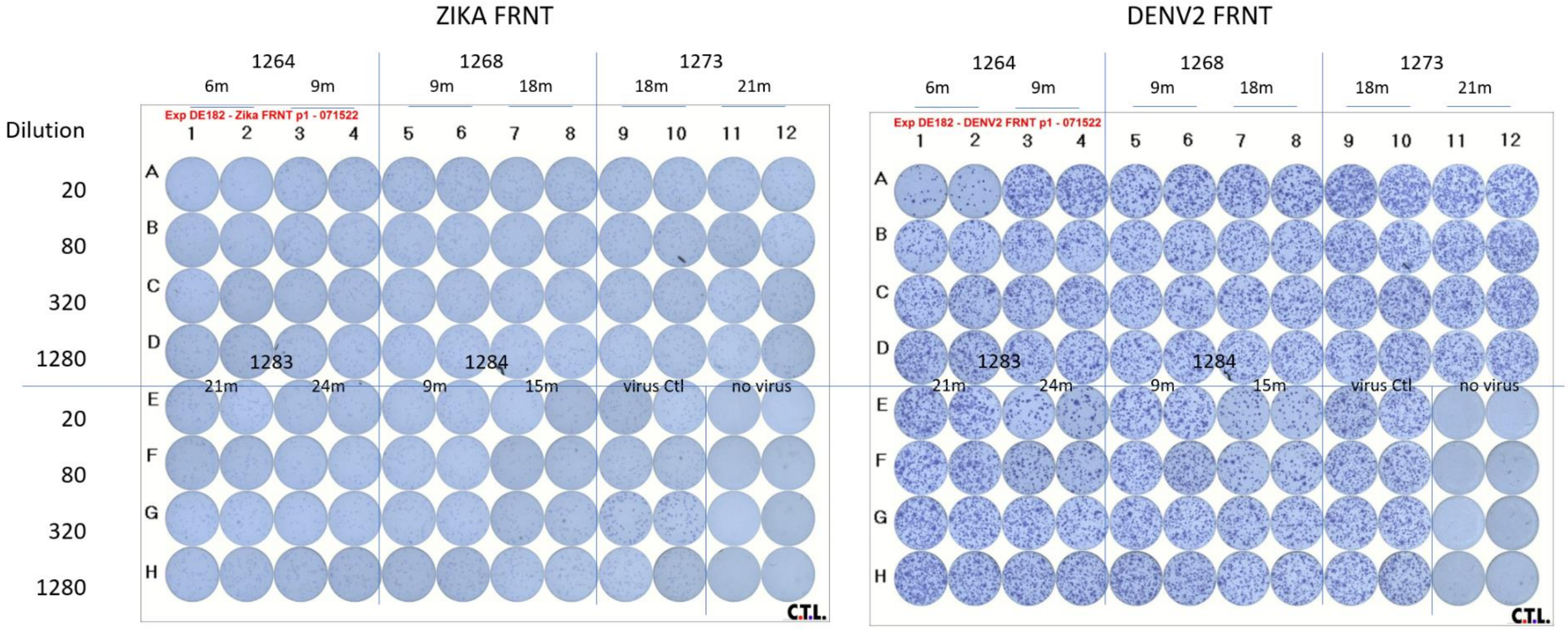

ZIKA FRNT
DENV2 FRNT
1264
1268
1273
6m
9m
18m
21m
Dilution
20
80
320
1280
1283
1284
24m
15m
virus Ctl
no virus
Exp DE182 - Zika FRNT p1 - 071522
Exp DE182 - DENV2 FRNT p1 - 071522
C.T.L.

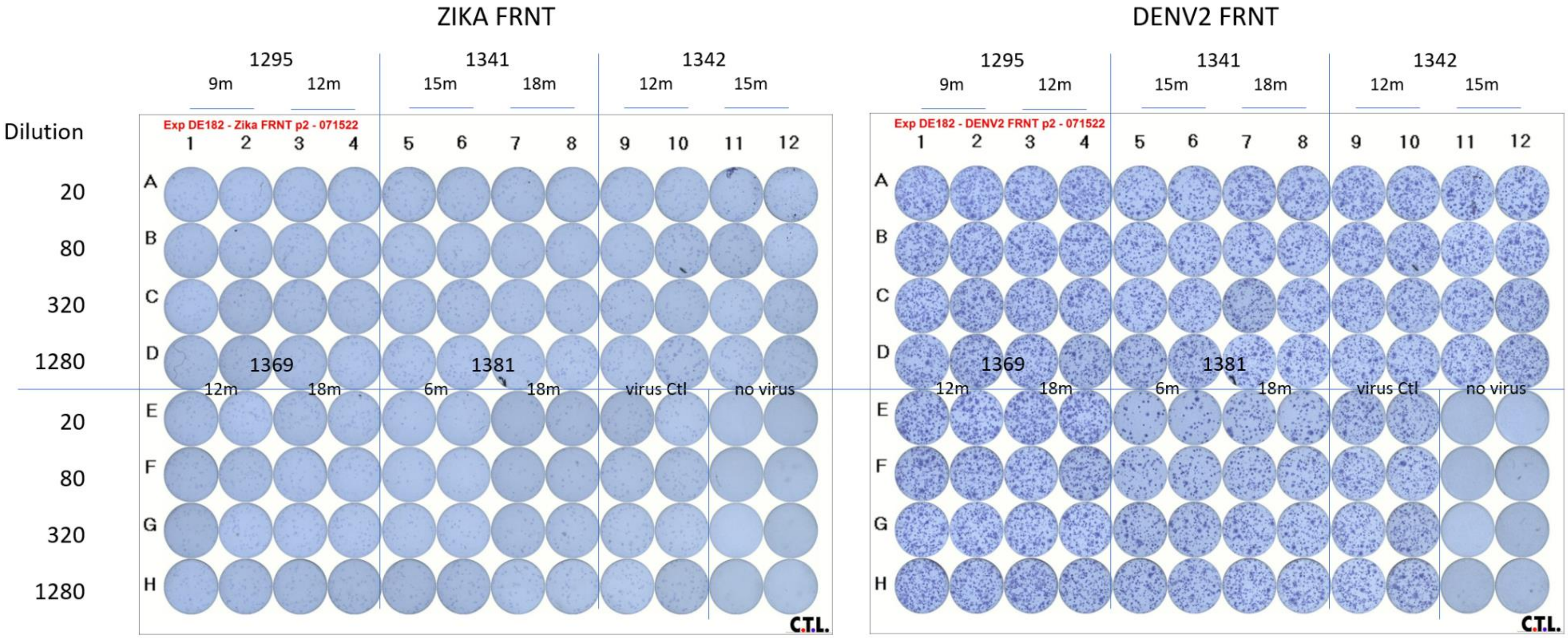
ZIKA FRNT
DENV2 FRNT
1295
9m
12m
1341
15m
18m
1342
12m
15m
Dilution
20
80
320
1280
1369
12m
18m
1381
6m
18m
virus Ctl
no virus
20
80
320
1280
Exp DE182 - Zika FRNT p2 - 071522
Exp DE182 - DENV2 FRNT p2 - 071522
1 2 3 4 5 6 7 8 9 10 11 12
A B C D E F G H
C.T.L.

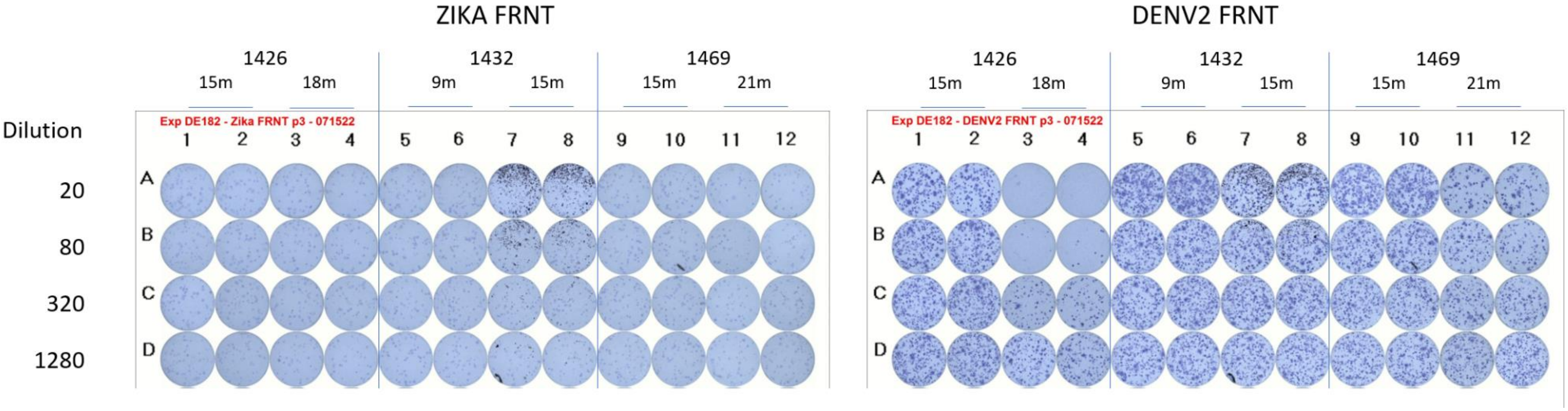
ZIKA FRNT
DENV2 FRNT
1426
15m
18m
1432
9m
15m
1469
15m
21m
Dilution
20
80
320
1280
Exp DE182 - Zika FRNT p3 - 071522
Exp DE182 - DENV2 FRNT p3 - 071522
1 2 3 4 5 6 7 8 9 10 11 12
A B C D

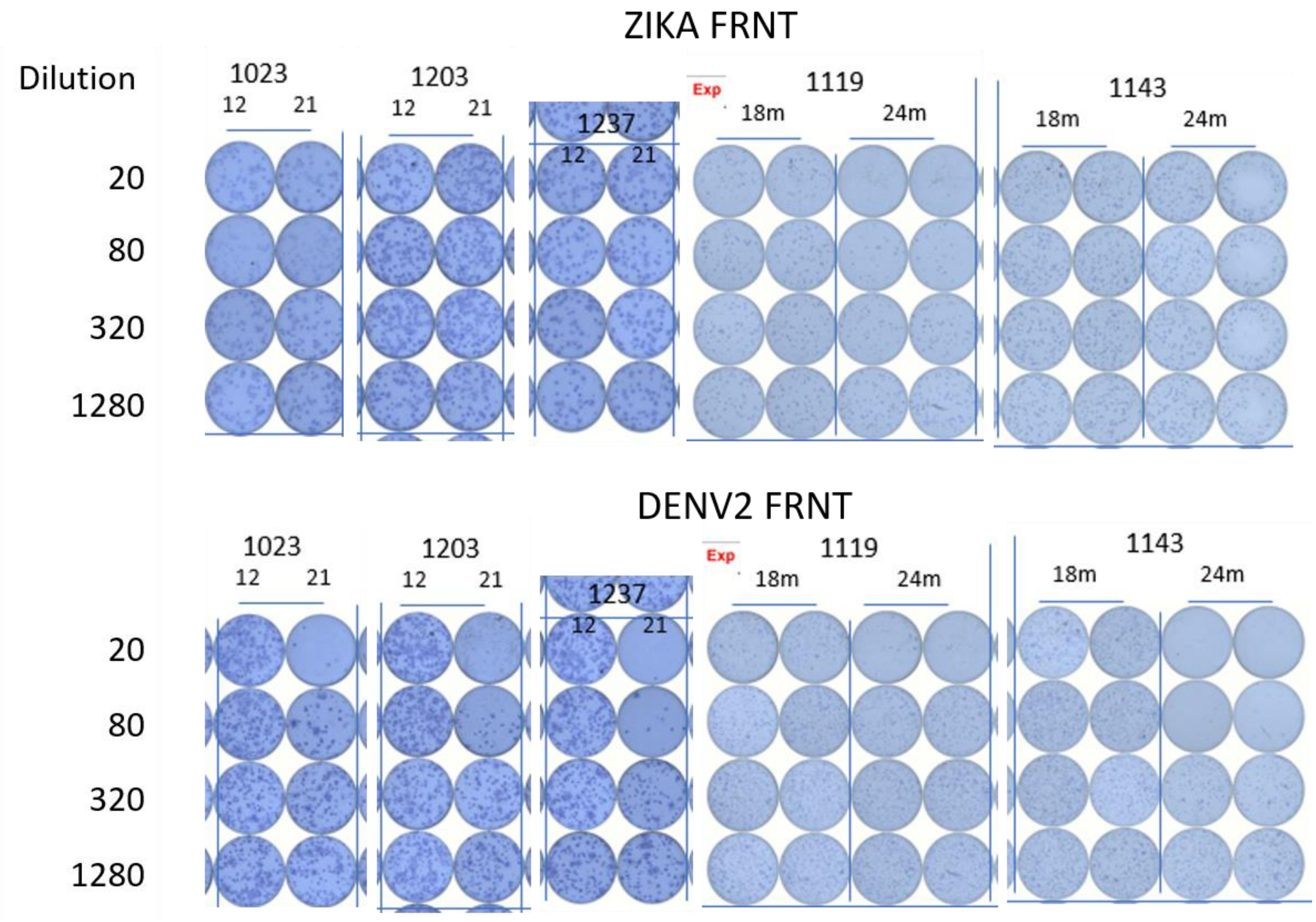
ZIKA FRNT
Dilution
20
80
320
1280
1023
12 21
1203
12 21
1237
12 21
Exp
1119
18m 24m
1143
18m 24m
DENV2 FRNT
20
80
320
1280
1023
12 21
1203
12 21
1237
12 21
Exp
1119
18m 24m
1143
18m 24m
